# Diverticular disease of the colon presenting as pyometra: a case report

**DOI:** 10.1186/1752-1947-8-135

**Published:** 2014-05-04

**Authors:** Susmita Pankaja, Astrit Rrukaj, Uma Bathula

**Affiliations:** 1 Department of Obstetrics & Gynaecology, Mid Staffordshire NHS Foundation Trust, Stafford Hospital, Weston Road, Stafford ST16 3SA, UK; 2 Department of Obstetrics & Gynaecology, University Hospital of North Staffordshire, Princes Road, Stoke-on-Trent ST4 6QG, UK

**Keywords:** Pyometra, Diverticular abscess, Endometrial cancer

## Abstract

**Introduction:**

Pyometra can be caused by various etiologies. We present a rare case of diverticular disease of the colon presenting as pyometra. This type of presentation can be challenging even for an astute clinician.

**Case presentation:**

A 74-year-old Caucasian woman with a history of pyometra was referred to our gynecology clinic as an urgent case. She was obese, diabetic and hypertensive. Due to the patient profile and the clinical presentation, clinicians were misled toward a diagnosis of possible endometrial cancer. After further investigations, she was found to have colouterine fistula secondary to a diverticular abscess of the sigmoid colon.

**Conclusions:**

Persistent vaginal discharge due to pyometra can be caused by diverticular disease of the colon. Clinicians should be aware of this important differential diagnosis.

## Introduction

Diverticular disease of the bowel commonly affects the sigmoid colon. Symptoms can range from mild to acute bouts of diverticulitis complicated by abscess or frank perforation. Colouterine fistula is an uncommon complication of diverticular abscess, which can present as pyometra and clinically mimic endometrial cancer.

## Case presentation

A 74-year-old Caucasian woman was referred to our gynecology clinic for suspected pyometra. She was obese, diabetic and hypertensive. Outpatient hysteroscopy revealed a thickened white endometrium and pyometra. A pipelle endometrial sample was taken and sent for histology.

Three days later, she presented to the accident and emergency department feeling unwell, with profuse foul-smelling vaginal discharge and bleeding per rectum. She was admitted and started on intravenous antibiotics for suspected uterine sepsis.

The pipelle biopsy showed pus cells only. Her carcinoembryonic antigen (CEA) level was normal and CA-125 slightly raised at 73. Vaginal swabs showed no pathogens. As the clinical picture was highly suggestive of endometrial cancer, a magnetic resonance imaging (MRI) scan was performed, which revealed pyometra but no obvious tumor.

A repeat hysteroscopy was done under general anaesthetic and drainage of pyometra was performed. About 50ml of pus was drained and an endometrial biopsy was taken. Pus culture showed mixed anerobic growth and the repeat endometrial biopsy revealed acute on chronic endometritis. She improved clinically with intravenous antibiotics and was discharged home.

She presented 10 days later to the accident and emergency department with a history of collapse, acute abdominal pain, diarrhoea, vomiting and bleeding per rectum. A contrast computed tomography (CT) scan of the abdomen requested by the general surgeons was unremarkable. A sigmoidoscopy and colonoscopy revealed significant diverticular disease with prominent thickened folds. She improved with conservative management.

She presented to the surgical team three months later with acute abdominal pain. A CT scan was repeated, which revealed an inflamed thick-walled sigmoid colon that tracked down to the uterus (a colouterine fistula). Our patient underwent laparotomy where a large diverticular mass was seen perforating into the uterus (a colouterine fistula) and adherent to its posterior wall. Pus was draining through the vagina. A Hartmann’s operation with total abdominal hysterectomy and bilateral salpingo-oophorectomy was performed.

She made a slow but complete recovery. At follow-up six months later she was asymptomatic. Our patient is now deceased but her death occurred due to a cause unrelated to the condition mentioned.

## Discussion

Pyometra can be caused by a number of gynecological conditions, both benign and malignant, leading to cervical stenosis [1-3]. Causes include puerperal infections, endometrial polyps, leiomyomas, and cervical stenosis due to surgery, radiotherapy or congenital cervical anomalies [2,3].

Yildizhan *et al*. suggested that the most common cause of pyometra is genital tract malignancy and treatment by radiotherapy [4]. However, in one report only 14 percent of pyometra was caused by malignancy [5]. Spontaneous perforation of pyometra, though rare, has been reported when pyometra occurs in association with malignancy [3,4]. Our patient’s profile suggested higher risk for endometrial cancer (overweight, hypertensive and diabetic). Hence, the clinical presentation of pyometra misled clinicians toward a diagnosis of endometrial cancer in the first instance.

The important differential diagnoses in these patients include gynecological malignancies (endometrial or cervical) and diverticular disease [1-5]. Other possible differential diagnoses for a patient with pyometra include cervical stenosis (commonly, postradiation), puerperal infections, endometrial polyps and leiomyomas [1-5].

Our patient had multiple admissions and investigations to find out the cause of her pyometra. A hysteroscopy and a pipelle biopsy were done on initial presentation. In view of her presentation with abdominal pain with rectal bleeding, she was evaluated by general surgeons and a contrast CT scan and an MRI scan were performed. Only when a repeat contrast CT was arranged three months later, was the correct diagnosis of colouterine fistula made.

Diverticular disease of the bowel commonly affects the sigmoid colon [1]. Symptoms can range from mild to acute bouts of diverticulitis complicated by abscess or frank perforation [2]. Colouterine fistula is a rare complication of diverticular disease of the colon [1]. In one case series, only 3 percent of internal fistulas caused by diverticular disease were colouterine fistula [1]. A colouterine fistula secondary to diverticulitis was first reported in 1929 [5,6]. Mandato *et al*. reported a case of a colouterine fistula at the level of the isthmus diagnosed at hysteroscopy [6]. The fistula was missed in our case during hysteroscopy due to a poor view in the presence of profuse amounts of pus.

Diagnosis depends on a high index of suspicion. It should be suspected in any patient with a persistent vaginal discharge and diverticulitis of the sigmoid colon. Presence of air and fluid within the uterus on ultrasound or CT scan in patients with diverticulitis or diverticular abscess is highly suggestive of a fistulous communication [1-3]. Imaging modalities may show evidence of communication between the uterus and colon, but may fail to demonstrate the fistulous tract [6-8]. In our patient, the first CT scan did not reveal any fistulous tract. Resection anastomosis in one stage can be curative [1,8]. Other surgical options include resection and diversion or a Hartmann procedure [1].

## Conclusions

Gynecological malignancy is a common cause of pyometra and can mislead clinicians. Persistence of pyometra with a negative endometrial biopsy should prompt investigations for presence of a fistulous tract especially in the presence of known diverticular disease of the colon. Colouterine fistula, though a rare complication of diverticular disease of the colon, can masquerade as pyometra.

## Consent

All reasonable and exhaustive attempts to contact our deceased patient's next of kin failed. This case report contains no patient-identifiable features, and no images were included.

## Competing interests

The authors declare that they have no competing interests.

## Authors’ contributions

SP, AR and UB were involved in the management of our patient. SP collected the data/details and prepared the manuscript, revised it and submitted it. AR collected the data/details and prepared the manuscript. UB prepared and revised the manuscript. All authors read and approved the final manuscript.
